# Experimental Evidence that Stochasticity Contributes to Bacterial Composition and Functioning in a Decomposer Community

**DOI:** 10.1128/mBio.00568-19

**Published:** 2019-04-16

**Authors:** Michaeline B. N. Albright, Alexander B. Chase, Jennifer B. H. Martiny

**Affiliations:** a Dept. of Ecology and Evolutionary Biology, University of California, Irvine, Irvine, California, USA; University of California, Berkeley; University of Oklahoma; University of Uppsala

**Keywords:** beta-diversity, dispersal, ecological drift, ecosystem functioning, stochastic

## Abstract

Stochasticity emerging from random differences in replication, death, mutation, and dispersal is thought to alter the composition of ecological communities. However, the importance of stochastic effects remains somewhat speculative because stochasticity is not directly measured but is instead inferred from unexplained variations in beta-diversity. Here, we performed a field experiment to more directly disentangle the role of stochastic processes, environmental selection, and dispersal in the composition and functioning of a natural bacterial decomposer community in the field. To increase our ability to detect these effects, we reduced initial biological and environmental heterogeneity using replicate nylon litterbags in the field. We then applied two treatments: ambient/added precipitation and bacterial and fungal dispersal using “open” litterbags (made from 18.0-μm-pore-size mesh) (“open bacterial dispersal”) versus bacterial and fungal dispersal using “closed” litterbags (made from 22.0-μm-pore-size mesh) (“closed bacterial dispersal”). After 5 months, we assayed composition and functioning by the use of three subsamples from each litterbag to disentangle stochastic effects from residual variation. Our results indicate that stochasticity via ecological drift can contribute to beta-diversity in bacterial communities. However, residual variation, which had previously been included in stochasticity estimates, accounted for more than four times as much variability. At the same time, stochastic effects on beta-diversity were not attenuated at the functional level, as measured by genetic functional potential and extracellular enzyme activity. Finally, dispersal was found to interact with precipitation availability to influence the degree to which stochasticity contributed to functional variation. Together, our results demonstrate that the ability to quantify stochastic processes is key to understanding microbial diversity and its role in ecosystem functioning.

## Abstract (precis)

Randomness can alter the diversity and composition of ecological communities. Such stochasticity may therefore obscure the relationship between the environment and community composition and hinder our ability to predict the relationship between biodiversity and ecosystem functioning. This study investigated the role of stochastic processes, environmental selection, and dispersal in microbial composition and its functioning on an intact field community. To do this, we used a controlled and replicated experiment that was similar to that used to study population genetics in the laboratory. Our study showed that, while the stochastic effects on taxonomic composition are smaller than expected, the degree to which stochasticity contributes to variability in ecosystem processes may be much higher than previously assumed.

## INTRODUCTION

The fields of evolutionary biology and ecology share a central question about biodiversity: what are the relative levels of impact of deterministic forces and stochastic forces? In evolution, the debate centers on the contribution of random genetic drift, a stochastic force, in shaping within-species diversity (1). In ecology, a similar question applies, but the area of inquiry shifts from the population level to the community level: what is the impact of ecological drift—variability emerging from random differences in replication, death, mutation, and dispersal among individuals (2–4)—in shaping community structure?

Numerous studies have suggested that stochasticity influences beta-diversity (compositional variation among communities) in plant and animal communities (5, 6), as well as microbial communities (7–12). Such studies have often tracked changes in composition across spatial and temporal gradients while measuring numerous biotic and abiotic variables (13–17). Although they do not measure stochasticity, they apply statistical variance partitioning to infer the importance of stochastic processes (18–20). Alternative approaches compare observational or experimental data to null models (12, 14, 21) or to neutral theory process models (22).

While no one approach is perfect, experiments that attempt hold environmental conditions constant in well-replicated experimental designs offer a valuable method to directly assess the importance of stochasticity (11). Indeed, evolutionary biologists have long measured the effect of genetic drift on population diversity by using highly controlled laboratory experiments (23, 24). Similar experiments aiming to quantify the effect of ecological drift (or of stochastic processes more generally) on community diversity, particularly under natural field conditions, are lacking. Those field studies that have been performed (see, e.g., references 6, 25, 26, and 27) typically focused on the dynamics of initial community assembly or manipulate stochasticity directly (e.g., by altering initial colonization). Thus, those studies primarily addressed the role of priority effects of introducing stochasticity to communities; however, experiments that measure stochasticity arising through ecological drift are still needed.

Microorganisms are also highly relevant for ecosystem functioning, and the importance of stochasticity ultimately depends on whether this compositional variation translates into variation in functioning. By one argument, stochastic variation among microbial communities would attenuate at the ecosystem level, because many taxa perform common functional processes (e.g., (17, 28). Alternatively, this variation might translate into functional differences, particularly for processes performed by a limited number of taxa (29). Indeed, the functional effects of stochastic variation have been observed for microbial communities in bioreactors (30), on decomposing wood (25), and in floral nectar (26).

Here, we aimed to experimentally quantify the influence of stochasticity on the composition and functioning of a highly diverse decomposer community under natural field conditions. Microbial communities in leaf litter offer particularly tractable systems to perform such experiments. In particular, intact (already assembled) communities can be “extracted” from their environment, homogenized, and then reinoculated into litterbags containing a sterile, homogenized litter substrate. This approach minimizes initial biological and environmental heterogeneity within and between litterbags, improving our ability to detect stochastic variation. In addition, because the initial communities are so abundant (∼10 million individuals), we can assume that the stochasticity that arises occurs primarily through ecological drift rather than priority effects. Further, the metrics of functional processes can be easily measured on the litterbags, including functional gene composition, activity of extracellular enzymes, litter chemistry, and litter mass loss. Indeed, the composition of microbial decomposers is known to influence the rate and quality of plant litter decomposition (i.e., ecosystem function) at our study site (31, 32) and in other systems (33, 34).

We also investigated if the rate of microbial dispersal altered the degree of stochastic effects by adjusting the mesh size of the litterbags, to allow or block the bacterial immigration into established decomposer communities. The role of dispersal in the balance of deterministic versus stochastic processes is particularly complex, as it may contribute to stochastic or deterministic variation. On the one hand, dispersal rates are thought to moderate the role of ecological drift (3, 4), with higher migration between locations homogenizing species composition among locations (reducing beta-diversity). On the other hand, dispersal may contribute to deterministic differences in community composition if taxa differ in their dispersal abilities.

**FIG 1:**
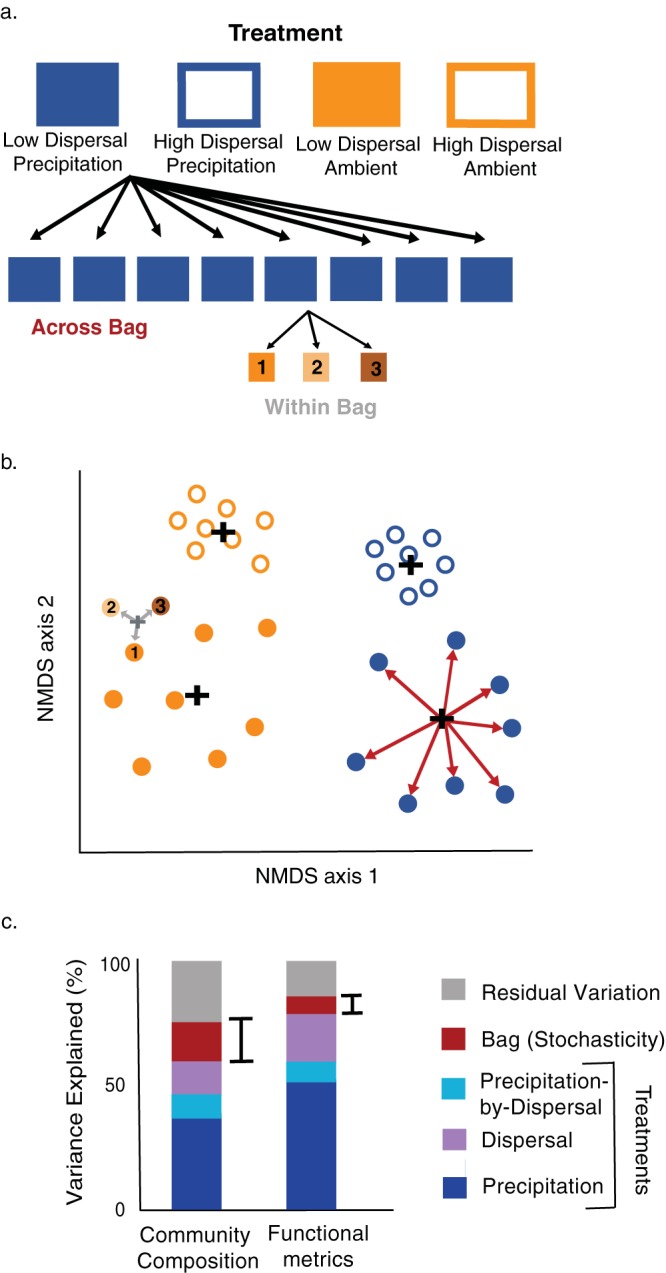
(a) Schematic diagram of experimental design. Four treatments were replicated eight times for a total of 32 litterbags containing homogenized leaf litter communities on a common litter substrate. To account for spatial heterogeneity within a litterbag, we assayed compositional and functional metrics on three subsamples from each of the litterbags. (b) Ordination showing hypothesized variation in community composition. Black crosses indicate group centroids of the different treatments and distances between the black crosses reflect variation among treatments. Within-treatment variation is denoted by red arrows. This variation was calculated after accounting for within bag variation (shown here for just one bag denoted by gray arrows and gray centroid). (c) Hypotheses for the relative variations in community composition and ecosystem function metrics. Variations due to the treatments (precipitation [blue], dispersal [purple], and a precipitation-by-dispersal interaction [light blue]) correspond to the distances between the centroids indicated in panel b. Variations due to stochasticity (red) correspond to the red arrows in panel b. Similarly, residual variations (gray) correspond to the gray arrows in panel b.

Finally, we manipulated precipitation, a factor that is known to influence microbial composition in this system (35). This treatment was included to provide deterministic variation against which we could compare any stochastic variation that we could detect. Thus, our experimental design included two crossed factors: dispersal (i.e., bacterial and fungal dispersal using “open” litterbags [made from 18.0-μm-pore-size mesh] [“open bacterial dispersal”] versus bacterial and fungal dispersal using “closed” litterbags [made from 22.0-μm-pore-size mesh] [“closed bacterial dispersal”]) and precipitation (ambient versus added water) (Fig. 1a). We further disentangled the effects of stochasticity (among-bag variation across treatment replications) from within-bag variation (residual variation due to within-bag spatial heterogeneity and technical error) by assaying all metrics on three subsamples from each litterbag replicate (Fig. 1a). While spatial heterogeneity within a litterbag might itself be due to both deterministic and stochastic forces, it could erroneously contribute to beta-diversity among litterbags, our spatial scale of interest in this experiment (15, 36). To account for this, we assayed compositional and functional metrics on three subsamples from each of the litterbags. With this design, we directly tested for significant compositional differences among litterbags within the treatments, allowing us to quantify the influence of stochasticity on beta-diversity.

This experimental design allowed us to test three main hypotheses as follows. In hypothesis 1 (H1), a detectable amount of beta-diversity among litterbag communities is due to stochasticity (quantified by the red arrows in Fig. 1b, which correspond to the red bars in Fig. 1c); in H2, stochastic variation is higher for community composition than for functional processes (comparison of red bars in Fig. 1c); and in H3, reduced dispersal increases the effect of stochasticity (spread of open versus closed circles in Fig. 1b). Altogether, our results suggest that stochasticity likely contributes less to the beta-diversity of intact microbial communities than previous studies estimated; however, this stochasticity translates into a measurable degree of functional variation in this decomposer community.

## RESULTS

### Overall treatment effects.

Before addressing our three hypotheses, we first considered the overall levels of significance of the two treatments (dispersal and precipitation). (If the treatments did not significantly affect the compositional or functional metrics, then we assumed that they did not explain any variation and removed the factors from the variance calculations described below.) The dispersal and precipitation treatments had minimal effects on bacterial abundance and alpha-diversity. The average bacterial abundance, measured by cell densities, was ∼9 × 10^8^ cells per gram of dry litter, and this density did not vary across treatments (analysis of variance [ANOVA]; *P* > 0.05) (see Fig. S4a in the supplemental material). Fungal abundances, as assessed by hyphal counts, also did not differ (*P* > 0.05; Fig. S4b). As with the abundance data, the numbers of OTUs (operational taxonomic units) observed by 16S rRNA amplicon sequencing did not differ significantly among treatments (*P* > 0.05; Fig. S5a), but the numbers were slightly lower than the level of richness observed (1,002 ± 94 OTUs) in the initial inoculum. Diversity (Shannon diversity index) was significantly higher in the open bags than in the closed bags (dispersal; *P* = 0.049; Fig. S5b), and the level of diversity was lower in all treatments than in the inoculum.

**FIG 2:**
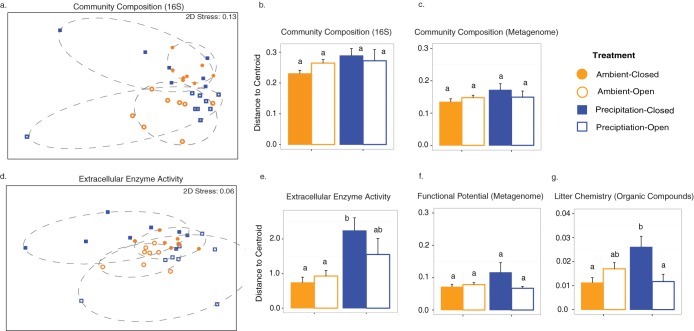
(a and d) Nonmetric multidimensional scaling (NMDS) ordinations showing variability in (a) 16S bacterial community composition (Bray-Curtis dissimilarity) and (d) extracellular enzyme activity (Euclidean distance). Each point represents the median of data determined from the 3 replicates from the bag (*n* = 32). 2D, two dimensional. (b, c, e, f, and g) Within-group distances ± standard errors (SE) for (b) 16S bacterial community composition (Bray-Curtis dissimilarity), (c) metagenome bacterial community composition, (e) extracellular enzyme activity (Euclidean distance), (f) functional genetic potential, and (g) organic litter chemistry of replicate litterbags within treatments (Ambient-Closed, Ambient-Open, Precipitation-Closed, Precipitation-Open). The individual sample points used to calculate within group distances are shown in the NMDS plots in panels a and b (see also Fig. S7a and Fig. S9).

In contrast to its minor effect on alpha-diversity, dispersal significantly altered bacterial beta-diversity levels among litterbags as assessed by 16S rRNA gene sequencing (Fig. 2a) (permutational multivariate analysis of variance [PERMANOVA]; *P* = 0.001) (see Table S1a in the supplemental material). (Note that for the possible outliers indicated in Fig. 2a, the composition within these bags was highly consistent across the three subsamples [Fig. S2].) Finally, the added precipitation did not alter the composition overall (Table S1a), but the effect of dispersal on composition depended on the precipitation treatment (a significant dispersal-by-precipitation interaction; *P* = 0.029). Similar results were observed whether composition was assessed by 16S rRNA gene sequencing or metagenomic sequencing (Fig. S6), although in the latter case, the dispersal-by-precipitation interaction was not statistically significant (Table S1b).

10.1128/mBio.00568-19.10 TABLE S1 Nested permutational multivariate analysis of variance (MANOVA) for (a) bacterial community composition (16S amplicon sequencing), (b) bacterial community composition from metagenomic data (marker genes), (c) functional composition from metagenomic data (Pfam protein families), and (d) extracellular enzyme activity (EEA) composition. The fag was nested within the precipitation and dispersal treatments, and significant differences among bag replicates indicate the influence of stochastic effects. Download Table S1, DOCX file, 0.02 MB. Copyright © 2019 Albright et al. 2019 Albright et al. This content is distributed under the terms of the Creative Commons Attribution 4.0 International license.

### Stochasticity influences taxonomic composition.

**FIG 3:**
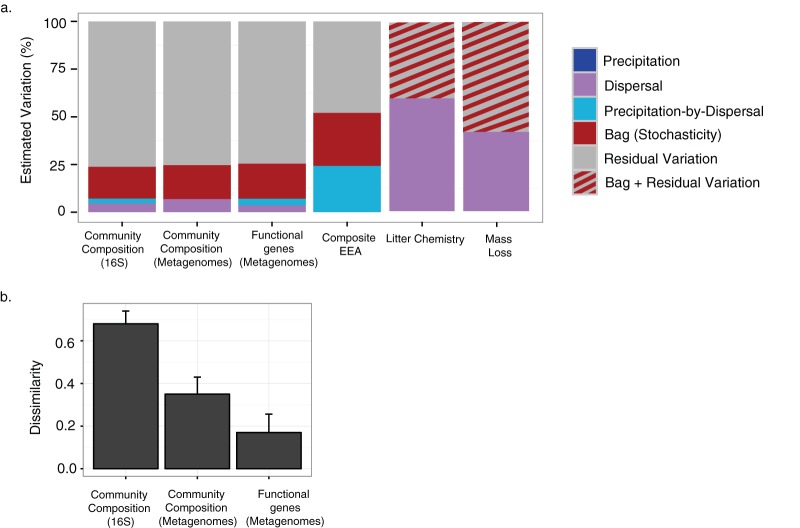
(a) Percentages of estimated variation across a variety of community composition and ecosystem function metrics explained by the environment, dispersal, environment-by-dispersal, across-replicate bag data (stochasticity), and residual variation. For community composition and extracellular enzyme activity, estimates were derived from a PERMANOVA model; for mass loss and litter chemistry, estimates were derived from a two-way ANOVA model. (b) Average Bray-Curtis dissimilarity values ± standard deviations (SD) from comparisons between samples for 16S and metagenomic sequence data.

The compositions of the initial inoculum samples were more similar (average Bray-Curtis [BC] dissimilarity = 0.50 ± 0.01) and beta-diversity increased over the course of the experiment within each of the treatments (average dissimilarity ranged from 0.62 ± 0.03 to 0.69 ± 0.06 within the four treatments) (Fig. S2). After accounting for treatment effects, the compositions differed significantly among the litterbag replicates at the end of the experiment (*P* = 0.001; Table S1), supporting our first hypothesis (that stochasticity contributes to beta-diversity). Although the dispersal treatment data were highly statistically significant, this effect explained a relatively small proportion (5%) of the total compositional variation among bags (Fig. 3a). The dispersal-by-precipitation interaction explained even an even smaller amount (2.5%) of the variation. In contrast, variability among replicate litterbags within a treatment—the component that estimates stochastic variation among bags—accounted for 16.5% of the total variation, or more than twice as much as all treatment effects combined (Fig. 3a). Similar trends were observed in assays of taxonomic composition performed through metagenomic sequencing (Fig. 3a). Thus, regardless of the method used to assay taxonomic composition, stochasticity contributed significantly to beta-diversity among the litterbags. The estimates of variation presented above are reported as relative proportions, but it is also revealing to consider the overall beta-diversity across samples observed for each metric. The average level of taxonomic dissimilarity between any two samples (Bray-Curtis index) for the community assayed by 16S rRNA gene sequencing was twice as high as the level seen with metagenomic sequencing (Fig. 3b).

### Stochasticity influences functional processes.

In contrast to our second hypothesis, the degree of stochasticity observed did not appear to show attenuation at the functional level. To test this, we assayed differences in microbial functioning between the litterbags by examining four factors: (i) the genetic functional potential of the communities (analyzed via metagenomics); (ii) extracellular enzyme activities (EEA); (iii) litter chemistry (which might vary if the communities decomposed different chemical fractions of the leaf litter); and (iv) total mass loss. In analysis of functional potential (assayed by metagenomic sequencing), stochasticity accounted for 18% of the variation, while only 7% was due to the treatments (Fig. 3; see also Table S1c). A similar fraction of variability was attributed to stochastic effects in analyses of only functional genes involved in either carbohydrate degradation (i.e., glycoside hydrolases and carbohydrate binding modules) or the nitrogen cycle (Fig. S7). As we would expect, however, the total variation in functional gene composition was lower than for either metric of taxonomic composition; the average level of Bray-Curtis dissimilarity determined for the functional metric was a quarter that for the 16S data and half that for the metagenomic taxonomic data (Fig. 3b).

Stochasticity accounted for an even greater degree of variation (28%) in EEA composition among bags (Fig. 3a). For this metric, the precipitation and dispersal treatments also influenced EEA composition strongly through an interactive effect, explaining 24% of the observed variation (PERMANOVA: *P* = 0.007) (Fig. 2d; see also Table S1d). This effect was driven by higher enzymatic activity in the open-ambient and closed-precipitation treatments for most of the individual enzymes (Fig. S8).

Because we could not take within-bag subsamples for litter chemistry and mass loss, we cannot separate the contributions of stochasticity (between replicate bag differences) and residual variation to these functional metrics. Combined, however, these effects contributed to 40.6% of the variation in litter chemistry and 58.5% of the variation in mass loss (Fig. 3a). The dispersal treatment accounted for 59.4% and 41.5% of the remaining variation, respectively. Open litterbags lost more mass than closed litterbags (ANOVA: dispersal; *P = *0.002, Fig. S4c), and the composition of the organic compounds in the plant litter differed between the open and closed bags (PERMANOVA: dispersal; *P* = 0.001, Fig. S9). Relative to the initial litter composition, the fraction of hemicellulose (e.g., xylan) increased in the open bags, while the fraction of more recalcitrant carbohydrates (i.e., lignin) increased in the closed bags (Fig. S9).

### Minor impacts of dispersal on stochasticity.

Overall, reduced dispersal did not increase the stochasticity of the decomposer community or its functioning, in opposition to our third hypothesis. However, there was evidence that this result may depend on precipitation. Levels of beta-diversity within a treatment group (mean distance to the centroid) did not differ significantly between treatments, whether composition was assayed by 16S sequencing or metagenomic sequencing (pairwise comparisons of group mean dispersions [PERMDISP]; *P* = 0.818) (Fig. 2b and c). However, EEA in the closed-precipitation treatment displayed significantly more variability than in the ambient treatments (pairwise test: versus closed and open, *P* = 0.004 and *P* = 0.01, respectively) (Fig. 2d and e). Furthermore, for the organic litter chemistry, the data from the closed-precipitation treatment were also significantly more variable than the data from the closed-ambient or the open-precipitation treatments (*P* = 0.02 and *P* = 0.05, respectively) (Fig. 2g). The trends showing higher variability in the closed-precipitation treatment were not statistically significant but were qualitatively similar for genetic functional potential (Fig. 2f) and taxonomic composition (Fig. 2b and c).

## DISCUSSION

Adapting an approach from experimental evolution studies, we aimed to quantify the degree to which stochasticity via drift impacts bacterial decomposer communities under natural field conditions. In support of our first hypotheses, stochastic variation contributed significantly to community composition. Two key factors in our experimental approach allowed robust quantification of stochastic effects. First, we homogenized the initial communities and limited the environmental variability across replicate litterbags within each treatment. Second, we differentiated between variability among replicates and any residual variation by taking subsamples from each replicate litterbag. This approach allowed us to separate our estimate of stochasticity from within-bag spatial heterogeneity or technical error, both of which are likely particularly important for microbial communities. In particular, technical error could be introduced by undersampling as well as by the molecular methods (e.g., variability added during DNA extraction, PCR amplification, and sequencing) (37–39).

While our results support those reported from prior studies suggesting that stochasticity contributes to microbial community diversity, they also indicate that such studies may have overestimated its importance (5, 20). Most studies do not take variation within a sampling unit into account in assessing community structure (40). However, residual variation, which is typically assigned to stochastic variation, accounted for three-quarters of the beta-diversity observed in our study. In contrast, our estimate of stochasticity was dramatically lower (<20%) and necessarily represents an upper bound, as it includes effects that are feasibly uncontrollable (environmental variation between replicates across the square-meter [m^2^] study) and that are not stochastic but are highly unpredictable (i.e., chaos [41, 42]).

In contrast to our second hypothesis, stochasticity did not appear to be attenuated at the functional level. Indeed, the levels of stochastic variation in both genetic functional potential and EEA activity were similar to, if not higher than, those quantified for composition. Moreover, while we could not distinguish between stochasticity and residual variation for mass loss and litter chemistry, we expect that technical error associated with the functional measurements should account for a lower fraction of residual variation than technical error associated with taxonomic metrics (the latter measurements involve many fewer procedural steps). Furthermore, the mass loss measurement was made on the entire litterbag, eliminating variation due to spatial heterogeneity. Thus, we conclude that bacterial diversity generated through ecological drift can impact decomposer functioning, just as diversity maintained by niche-based, deterministic processes does (32).

Of course, spatial heterogeneity within replicates may be itself be driven by stochasticity or microscale environmental variation. Indeed, with appropriate technical methods, we could investigate the role of stochasticity in generating diversity at a smaller scale, such as between leaf fragments. For this study, however, we aimed to compare stochastic variation in composition with that in functional metrics at the same spatial scale. We therefore focused on the litterbag unit, as functional processes are usually measured at this spatial scale, across several centimeters or the approximate size of a litterbag, soil core, or gas flux collar.

It is also important that the relative influences of deterministic versus stochastic factors change under different conditions (14, 21), making direct quantitative comparisons to other studies problematic. Indeed, previous work at this site found significant effects of reduced precipitation on bacterial composition (31, 32), whereas adding precipitation here did not produce a significant main response, perhaps because of the length of the experiment or because of rapid evaporation occurring immediately after the water application treatment. In contrast, a study that sampled across a strong environmental gradient or among more-severe treatments would attribute more variation to deterministic effects and hence proportionally less to stochasticity and residual variation.

The distinction between relative variability and absolute variability is also key to interpreting the results of comparisons between the compositional and functional metrics. Consistent with previous studies (17, 28, 43), we observed that total variation across samples was lower for our functional metrics than for taxonomic composition at the genetic levels assessed here (Fig. 3b). In absolute terms, such a pattern is expected, because functional metrics are typically coarser (involving an aggregation of the activity of many taxa combined) than those for composition. However, we also found that the relative contributions of stochasticity stayed the same at both the community and function levels. The discrepancy between the relative effects and absolute effects highlights the need to distinguish these quantities in future studies.

Finally, reducing dispersal did not consistently increase stochastic effects on community composition or function. However, the third hypothesis was partially supported, as the dispersal treatment appeared to interact with the precipitation treatment to influence the degree of stochasticity in some of the functional metrics. We speculate that added precipitation allowed higher turnover (replication and death) and thus increased the degree of ecological drift over the duration of the experiment (44). Alternatively, cells from the added rainwater might have altered the composition directly. This result also agrees with recent theoretical work that suggested that the influence of dispersal on beta-diversity would depend on complex interactions with the environment (8, 36). We also cannot exclude the possibility that two other factors could have contributed to differences between and within the dispersal treatments. First, while the open bag mesh pore size was small (18 μm), very small grazers might have differentially colonized the open bags and not the closed bags. In addition, although we did not observe differences in water content between the open bags and the closed bags (see Materials and Methods), some slight variation in humidity might have occurred such that the environments differed between the treatments.

### Conclusions.

Our study data suggest that ecological drift measurably contributes to observed beta-diversity in mature bacterial communities and that this stochastic variation can translate into functional variability. A further issue is that of determining under which conditions stochastic processes become more or less influential (see, e.g., references 21 and 45). For instance, a recent hypothesis suggests that perturbations of animal microbiomes (from corals to humans) alter these communities in a stochastic manner (46). If, as observed here, this stochasticity translates into functional variability, then the functional consequences of such perturbations may also be stochastic. Hence, quantifying the role of stochastic processes in microbial communities is central to our ability to predict system functioning, whether such predictions are focused on the degradation of plant litter, on the health of the human gut, or on global biogeochemical cycling.

## MATERIALS AND METHODS

### Field experiment.

The grassland at Loma Ridge in Irvine, CA (33°44′N, 117°42′E, 365-m elevation), experiences a semiarid Mediterranean climate, with mean annual precipitation of 325 mm, most of which occurs between October and April, and is dominated by nonnative annual grasses (Bromus diandrus, Avena fatua) (47, 48). Surface leaf litter was collected in November 2014 from the field site and ground with a coffee grinder (KitchenAid model BCG111OB).

We constructed 32 litterbags (10 cm by 8 cm) from nylon mesh (Tisch International). To reduce heterogeneity within and between replicate bags, each litterbag was filled with mixed, ground, and sterilized litter that was reinoculated with a homogenized microbial community. For sterilization, the litter was autoclaved, wetted down with 0.9 M NaCl, left overnight, and autoclaved again. The litter (4.7 g) was then added to each nylon mesh litterbag, and each bag was sealed and gamma irradiated (>22 kGy). Initial chemistry measurements were performed on four litter samples (as described below) (see Fig. S1 in the supplemental material). We reinoculated all litterbags with 0.12 g (∼ 1.2 × 10^7^ cells) of ground and homogenized inoculum litter, freshly collected from the Loma Ridge grassland in January 2015, just prior to deploying the experiment. Community DNA from eight inoculum samples was sequenced (described below) to test the initial homogenization of the microbial community (Fig. S2).

10.1128/mBio.00568-19.1 FIG S1 Nonmetric multidimensional scaling (NMDS) ordinations showing variability in organic litter chemistry (Euclidean distance) for the inoculum and final samples. Download FIG S1, EPS file, 0.8 MB. Copyright © 2019 Albright et al. 2019 Albright et al. This content is distributed under the terms of the Creative Commons Attribution 4.0 International license.

10.1128/mBio.00568-19.2 FIG S2 Nonmetric multidimensional scaling (NMDS) ordinations showing variability in bacterial community composition (calculated using [a] Bray-Curtis [b] Raup-Crick, and [c] Jaccard dissimilarity analyses) with all replicate subsamples as well as initial inoculum subsamples plotted (black asterisks). We note that some interations of the Raup-Crick NMDS would not converge, because most of the dissimilarity values were near zero (see Materials and Methods). Download FIG S2, EPS file, 1.7 MB. Copyright © 2019 Albright et al. 2019 Albright et al. This content is distributed under the terms of the Creative Commons Attribution 4.0 International license.

Half of the bags (“open” litterbags) were made from 18.0-μm-pore-size mesh to allow the dispersal of bacteria and fungi into and out of the bags. The other half (“closed” litterbags) were made from 0.22-μm-pore-size mesh to limit bacterial and fungal dispersal. To test how the mesh size influenced water content, both litterbag types were deployed in a concurrent field experiment and collected weekly. The levels of water content of litter in the closed and open bags were not statistically significantly different (analysis of covariance [ANCOVA]; main effect of dispersal treatment [*F*_3.89_ = 0.40, *P* = 0.53]) (49). Further, the closed litterbags significantly reduce bacterial dispersal but do not remain completely sterile (49).

Inoculated litterbags were deployed on 8 January 2015 and collected on 5 June 2015, to coincide with most of the annual rains and litter decomposition. To minimize environmental variation, litterbags were placed in close proximity to one another (within a 1-m^2^ plot). To manipulate precipitation, we collected rainwater in December 2014 and stored it at 4°C until use. In the field, precipitation was manipulated by adding 120 ml of unsterilized rainwater to half of the open and closed litterbags at 5 time points during the 5-month duration of the experiment. This volume was equivalent to half of the rainfall of the first storm of the season, which was slightly higher than the rainfall of each subsequent storm during the experiment (Fig. S3). This application completely saturated the litter inside the bags. Upon collection, we weighed the litter remaining in each bag and dried a subsample at 60°C to obtain dry mass. Mass loss is reported as the percentage of loss of initial dry mass.

10.1128/mBio.00568-19.3 FIG S3 Precipitation at Loma Ridge between January 2015 and June 2015. Red lines indicate precipitation additions. Download FIG S3, EPS file, 0.6 MB. Copyright © 2019 Albright et al. 2019 Albright et al. This content is distributed under the terms of the Creative Commons Attribution 4.0 International license.

10.1128/mBio.00568-19.4 FIG S4 (a) Bacterial cell counts per gram of dry litter, (b) fungal hyphal lengths, and (c) mass loss across the four treatments. ANOVA results corresponding to treatment effects include precipitation effects (P), dispersal effects (D), precipitation-by-dispersal effects (PxD), and bag effects nested within the precipitation and dispersal treatments [B(PxD)]. Download FIG S4, EPS file, 1.3 MB. Copyright © 2019 Albright et al. 2019 Albright et al. This content is distributed under the terms of the Creative Commons Attribution 4.0 International license.

10.1128/mBio.00568-19.5 FIG S5 Treatment effects, including precipitation effects (P), dispersal effects (D), precipitation-by-dispersal effects (PxD), and bag effects [B(PxD), indicating that the bag is nested within the precipitation and dispersal treatments] on (a) bacterial richness (number of OTUs) and (b) bacterial diversity (Shannon diversity) as assayed by 16S rRNA gene sequencing, after rarefaction to standardize for sequencing effects among samples. Download FIG S5, EPS file, 1.1 MB. Copyright © 2019 Albright et al. 2019 Albright et al. This content is distributed under the terms of the Creative Commons Attribution 4.0 International license.

10.1128/mBio.00568-19.6 FIG S6 Relative abundances of bacterial families as determined by (a) 16S amplicon sequencing and (b) metagenomic sequencing found for each treatment type and the inoculum averaged across replicates. Download FIG S6, EPS file, 2.6 MB. Copyright © 2019 Albright et al. 2019 Albright et al. This content is distributed under the terms of the Creative Commons Attribution 4.0 International license.

10.1128/mBio.00568-19.7 FIG S7 Percentage of estimated variation explained by treatment effects, stochastic effects, and measurement error for (a) individual extracellular enzymes, including α-glucosidase (AG), acid phosphatase (AP), β-glucosidase (BG), β-xylosidase (BX), cellobiohydrolase (CBH), leucine aminopeptidase (LAP), and N-acetyl-β-d-glucosaminidase (NAG) and averages determined for those seven enzymes, and (b) for carbon and nitrogen cycling genes in the metagenomes. Download FIG S7, EPS file, 1.5 MB. Copyright © 2019 Albright et al. 2019 Albright et al. This content is distributed under the terms of the Creative Commons Attribution 4.0 International license.

10.1128/mBio.00568-19.8 FIG S8 Potential activities of seven extracellular enzymes, including α-glucosidase (AG), acid phosphatase (AP), β-glucosidase (BG), β-xylosidase (BX), cellobiohydrolase (CBH), leucine aminopeptidase (LAP), and N-acetyl-β-d-glucosaminidase (NAG). Measurements are shown for the inoculum (*n* = 8) and 3 replicates from each final litterbag (*n* = 96). Download FIG S8, EPS file, 1.3 MB. Copyright © 2019 Albright et al. 2019 Albright et al. This content is distributed under the terms of the Creative Commons Attribution 4.0 International license.

10.1128/mBio.00568-19.9 FIG S9 Percent change in individual organic fractions, including cellulose, hemicellulose, lignin, structural carbohydrates, and protein. Percent change was calculated as follows: (final fraction − initial fraction)/initial faction * 100. Download FIG S9, EPS file, 0.7 MB. Copyright © 2019 Albright et al. 2019 Albright et al. This content is distributed under the terms of the Creative Commons Attribution 4.0 International license.

### 16S rRNA gene sequencing and analysis.

All assays (except litter chemistry and mass loss) were performed on 8 inoculum samples and 96 final collection samples (4 treatments × 8 bags × 3 subsamples) for a total of 102 samples. For the DNA extraction, 0.05 g litter was collected and stored at −80°C in a freezer until extraction. DNA was extracted following the FastDNA spin kit for soil (MP Biomedicals, LLC) protocol, with the following two modifications: (i) after the addition of sodium phosphate and MT (MP Biomedicals) buffer, samples were subjected to a three freeze-thaw cycles by immersing the samples for 30 s in liquid nitrogen and 3 min in a 60°C water bath; (ii) bead-beating was done in a FastPrep FP120 instrument (Bio101, Vista, CA, USA) at 5.5 m s^−1^ for 45 s.

We subjected the V4-V5 region of the 16S rRNA gene to PCR amplification following the Earth Microbiome protocol (50) with the 515 forward primer (GTGYCAGCMGCCGCGGTAA) and 926 reverse primer (CCGYCAATTYMTTTRAGTTT) designed as described in reference 50 and modified as described in reference 51. Briefly, 5 μl of a 1:50 dilution of DNA (average, 2.95 ± 0.88 ng DNA) was added to 1 unit per reaction mixture of Hot Start *Taq* DNA polymerase (New England BioLabs, Inc.), 1× PCR Rxn buffer (−MgCl_2_) (Invitrogen), 1,200 μM MgCl_2_ (Invitrogen), 200 μM deoxynucleoside triphosphate (dNTP), 0.2 μM forward primer and 0.2 μM reverse primer, 200 mM bovine serum albumin (acetylated) (PROMEGA), and H_2_O to reach a final volume of 25 μl. Following an initial denaturation step at 94°C for 3 min, the PCR was cycled 35 times at 94°C for 45 s, 55°C for 30 s, and 68°C for 20 s, with a final extension at 68°C for 10 min (35, 50). We amplified each subsample in duplicate.

Amplified samples were pooled based on gel pictures, with 1.0, 2.0, and 3.0 μl added for strong, moderate, and weak bands, respectively, into a low-binding tube. After pooling, PCR products were cleaned using an Agencourt AMPure XP PCR purification kit (Beckman Coulter Inc., Indianapolis, IN, USA), following the standard manufacturer’s instructions. To isolate the target band, the cleaned PCR products were run on a Tris-acetate-EDTA (TAE) agarose gel at 80 V for 1 h, and the resulting DNA was gel extracted and purified using a standard Zymoclean gel DNA recovery kit (Zymo Research Corp.). PCR products were quantified (Qubit double-stranded DNA [dsDNA] HS assay; Invitrogen) and assessed for quality using a high-sensitivity DNA assay on an Agilent Bioanalyzer. Multiplexed products were sequenced on a single-lane flow cell at the University of California (UC) Davis DNA Technologies Core using a paired-end Illumina MiSeq platform.

Sequence data were processed using the QIIME (version 1.9.1) toolkit (52). Paired-end files were joined, and operational taxonomic units (OTUs) were picked at the 97% identity level using UCLUST (53) with the nearest-neighbor method. Taxonomy was assigned using SILVA v119 as the reference database (54). Using the computed OTU matrix, we generated a rarefied composition table, randomly drawing the lowest common number of sequences (*n* = 5,187) from each sample to create 100 tables. To weigh rarer taxa more heavily, we transformed each table by taking the square root of each cell value and rounding to the nearest integer. We then calculated a median Bray-Curtis (BC) distance matrix, which was used in the remaining analyses (35). The average Shannon diversity level in all rarified OTU tables was also calculated (52).

We used the BC metric because we wanted to capture community variations in both relative abundance and richness that might arise during the experiment, whether that variation was caused by stochastic or deterministic forces. For comparison, we also considered how our results might change if we used a different dissimilarity metric. We thus calculated two additional metrics, the Jaccard metric and the Raup-Crick (RC) metric (using the raupcrick functions in vegan 2.4-2 [55]), for the same 100 rarefied OTU tables. All three metrics revealed similar patterns in compositional differences among the samples (Fig. S2). Indeed, the BC and Jaccard matrices were highly positively correlated (Spearman’s ρ = 0.98, *P* < 0.001 [Mantel test]), indicating that differences in relative abundances mirrored differences in the number of taxa shared among the litterbags. Similarly, the BC and RC matrices were also significantly correlated, albeit not as strongly (ρ = 0.49, *P* < 0.001). On the one hand, this weaker relationship might have been a consequence of the fact that RC is a richness-correcting metric. However, the richness levels did not vary among the treatments at the end of the study, with only a marginal precipitation-by-dispersal effect (Fig. S5). Instead, the lower correlation seems to have been due to the limited power of the RC metric to detect differences among samples with very high alpha-diversity, a limitation that was previously noted (55). As a result, 95% of the values in the Raup-Crick matrix were exactly the same and were assigned the lowest value possible. Indeed, the compositional differences between litterbags are very small by design; we homogenized the environment among the litterbags and inoculated with the same initial communities. Further, while the RC metric is recommended for studies aiming to disentangle stochastic versus determinic forces, we note that two previous studies (see, e.g., reference 12 and reference 14) tested their observations against a random null model. However, because patterns that look random can be caused to appear random by other mechanisms, our study did not assume a null model but instead used an experimental approach to hold the environment constant and to measure the influence of stochasticity directly.

### Metagenomic sequencing and analysis.

Metagenomic libraries were prepared using a Nextera XT DNA library preparation kit (Illumina, San Diego, CA, USA) and sequenced on an Illumina HiSeq 4000 system (150-bp paired ends) at the DNA Technologies Core, UC Davis, CA. Sequence data for metagenomics libraries were processed to obtain taxonomic and functional community composition. Merged paired-end reads (processed using PEAR [56]) and the remaining unmerged forward reads were combined and filtered using fastq-mcf in the EA-UTILS software package (57). Coding regions of the filtered reads were generated with FragGeneScanPlus (58).

Taxonomic compositions of metagenomic libraries were assigned using a custom reference genomic database (https://github.com/alex-b-chase/LRGCE), as previously described (59). Briefly, we used phylogenetic inference to search a subset of single-copy marker genes (60) against the reference database to generate taxonomic profiles for each sample. Functional profiles for each metagenomic library were obtained by searching translated reads using HMMER v3.1b2 (61) against the Pfam family database (62), where the top sequence matches with an E value of >e−04 were retained. Identity (ID) (taxonomic or pfam family)-by-sample matrices were converted to biom files, and tables were filtered to remove singletons. We rarefied the ID-by-sample matrices at even depths of 1,001 reads and 107,231 reads for the taxonomic and functional annotations, respectively. For rarefaction, 100 ID-by-sample matrices were randomly subsampled at even depths. Further processing in QIIME was performed using the same steps as were used with the 16S amplicon sequencing data. For pathway-specific analysis, genes involved in carbohydrate degradation (glycoside hydrolases and carbohydrate binding modules in Pfam [62] and the nitrogen cycle [63]) were extracted from the functional annotations to create pathway-specific ID-by-sample matrices. Here, the matrices were rarefied at even depths of 980 and 161, respectively. Further processing was performed as described for the overall functional genetic potential sequencing data.

### Extracellular enzymes.

We used spectrophotometric assays to characterize the potential extracellular enzyme activities (EEA) of the litter samples. Litter samples were stored at −80°C until processing. Sample homogenate preparation and flourimetric enzyme assays were performed using methods described previously in reference 64. We measured the potential activities of seven extracellular enzymes, including α-glucosidase (AG), acid phosphatase (AP), β-glucosidase (BG), β-xylosidase (BX), cellobiohydrolase (CBH), leucine aminopeptidase (LAP), and N-acetyl-β-d-glucosaminidase (NAG). We created a composite metric of the seven assays by calculating the Euclidean distance between the samples in each pairwise sample set after normalizing the measurements.

### Bacterial cell and fungal hypha length densities.

Bacterial cell densities were measured using flow cytometry, using a procedure modified from one described previously in reference 31. At sample collection, three 0.1-g subsamples of the litter were fixed with 5 ml of 1% phosphate-buffered glutaraldehyde solution within 6 h of collection. Fixed samples were stored in the dark at 4°C for up to 1 week. To extract cells from the litter, 0.55 ml of 0.1 mol/liter tetrasodium pyrophosphate was added to the sample and gently sonicated for 30 min in the dark at 4°C. The samples were then filtered through a 3.3-µm-pore-size syringe filter to remove large particulates. A 3-µl volume of SYBR green (200×) was added to 600 µl of sample and incubated in the dark at room temperature for 10 min. Stained particle counts were performed using flow cytometry (BD Accuri C6; BD Biosciences, San Jose, CA, USA). Each subsample was run three times on a flow cytometer. The flow cytometer was run for 2 min on medium speed, using a threshold value of 2,000. Gating parameters were optimized to count particle sizes in the size range of typical bacterial cells. Cell densities are reported as numbers of stained counts per g dry weight litter.

Fungal hyphal slides were prepared using a method described previously in reference 31. All slides were stored in the dark at 4°C prior to microscopy. Using a microscope (×100 magnification) and AxioVision Rel.4.5 software, 30 photographs were taken for each slide. The AxioVision ruler feature was used to outline and measure fungal hyphae. The average fungal hyphal length per gram of litter for each sample was calculated as follows: total hyphal length/(area of the photo × 30) × filter area × (0.1 g of litter/dilution factor).

### Litter chemistry.

Litter chemistry was assessed using near-infrared (IR) spectroscopy (Cumberland Valley Analytical Services, Hagerstown, MD). Relative amounts of organic compounds, including cellulose, hemicellulose, lignin, structural carbohydrates, and protein, were determined as fractions of the non-ash dried plant litter. A composite metric of the organic compounds was calculated using the Euclidean distance between the samples in each pairwise sample set. In addition, for each individual organic compound, we calculated the percentage of change (final fraction − initial fraction)/initial fraction × 100.

### Statistical analyses.

To test for differences among the treatments and to estimate the variance explained by each treatment, we performed a permutational multivariate analysis of variance (PERMANOVA) (PRIMER6 & PERMANOVA+; Primer-E Ltd., Ivybridge, United Kingdom) for the multivariate metrics. These models included precipitation and dispersal as main fixed factors, a precipitation-by-dispersal interaction, and bag replicates as a random nested factor within the precipitation and dispersal treatments, where significant differences among bag replicates indicated the influence of stochastic effects. For organic litter chemistry, the model did not include the nested factor, as we did not have subsamples. Analyses were run using type III partial sums of squares and a reduced model with 999 permutations (35, 65, 66). For the univariate metrics (e.g., richness and individual EEA), we used a factorial nested ANOVA design with precipitation and dispersal as fixed effects and also used a precipitation-by-dispersal interaction. For analysis of mass loss differences, we performed a standard two-way ANOVA (nonnested), because we did not have subsamples within the bags. The ANOVAs were conducted in the R software environment (67). We estimated the percentage of variation that could be attributed to each significant term for both the PERMANOVA (as described in reference 65) and the ANOVA (68).

The statistics determined as described above estimate variation across treatment types. To quantify the relative levels of variability within the treatment groups (i.e., the open-ambient, closed-ambient, open-precipitation, closed-precipitation groups), we measured the distance to the centroid within each treatment combination. For metrics with subsamples within bags, we first averaged the data corresponding to the three subsamples from each bag by finding the centroid of the subsamples using the “Distances among centroids” feature in PERMANOVA+ (65) such that each bag was represented by only one average measurement (*n *=* *32). Using a permutation test (999 permutations), we ran pairwise comparisons of group mean dispersions based on the four treatment groups (PERMDISP in PRIMER6 and PERMANOVA). A two-way model is not advised given difficulties in testing homogeneity of dispersions across multiple main effects (69).

### Data availability.

Metagenomic sequences are available through the MG-RAST server (https://www.mg-rast.org/mgmain.html?mgpage=project&project=mgp20922). Unprocessed metagenomic sequences are available through NCBI’s Sequence Read Archive (accession no. PRJNA414041). 16S rRNA sequences have been deposited in NCBI’s Sequence Read Archive (accession no. SRP119823).
