# Supplementary material for: Experimental Evidence that Stochasticity Contributes to Bacterial Composition and Functioning in a Decomposer Community
Source: mBio. 2019 Apr 16;10(2):e00568-19. doi: 10.1128/mBio.00568-19 (PMC6469972; doi:10.1128/mBio.00568-19)
Supplement: TABLE S1 [file mBio.00568-19-st001.docx]

**Table S1.** Nested permutational MANOVA for a) bacterial community composition (16S amplicon sequencing) b) bacterial community composition from metagenomic data (marker

genes) and c) functional composition from metagenomic data (Pfam protein families) d) extracellular enzyme activity (EEA) composition. Bag was nested within the precipitation and dispersal treatments, and a significant difference among bag replicates indicates the influence of stochastic effects.

a. **Community composition (16S)**

|  | df | SS | MS | Pseudo-F | P(perm) |
| --- | --- | --- | --- | --- | --- |
| Precipitation | 1 | 0.333 | 0.333 | 1.166 | 0.163 |
| Dispersal | 1 | 0.756 | 0.756 | 2.649 | **0.001** |
| Precipitation X Dispersal | 1 | 0.415 | 0.415 | 1.454 | **0.029** |
| Bag (Precipitation x Dispersal) | 28 | 8.418 | 0.293 | 1.605 | **0.001** |
| Residual | 59 | 10.67 | 0.181 |  |  |
|  |  |  |  |  |  |

b. **Community composition (metagenomes)**

|  | df | SS | MS | Pseudo-F | P(perm) |
| --- | --- | --- | --- | --- | --- |
| Precipitation | 1 | 0.11 | 0.11 | 1.38 | 0.11 |
| Dispersal | 1 | 0.27 | 0.27 | 3.48 | **0.001** |
| Precipitation X Dispersal | 1 | 9.17E-2 | 9.17E-2 | 1.17 | 0.226 |
| Bag (Precipitation x Dispersal) | 28 | 2.20 | 7.87E-2 | 1.69 | **0.001** |
| Residual | 61 | 2.84 | 4.66E-2 |  |  |
|  |  |  |  |  |  |

c. **Genetic functional potential (metagenomes)**

|  | df | SS | MS | Pseudo-F | P(perm) |
| --- | --- | --- | --- | --- | --- |
| Precipitation | 1 | 2.89E-2 | 2.89E-2 | 1.158 | 0.226 |
| Dispersal | 1 | 5.43E-2 | 5.43E-2 | 2.177 | **0.006** |
| Precipitation X Dispersal | 1 | 3.93E-2 | 3.93E-2 | 1.577 | **0.04** |
| Bag (Precipitation x Dispersal) | 27 | 0.69 | 2.56E-2 | 1.6813 | **0.01** |
| Residual | 55 | 0.8374 | 1.53E-2 |  |  |
|  |  |  |  |  |  |

**d. Potential extracellular enzyme activity**

|  | df | SS | MS | F | P |
| --- | --- | --- | --- | --- | --- |
| Precipitation | 1 | 2.7341 | 2.7341 | 0.25 | 0.89 |
| Dispersal | 1 | 23.316 | 23.316 | 2.13 | 0.111 |
| Precipitation X Dispersal | 1 | 58.728 | 58.728 | 5.37 | **0.007** |
| Bag (Precipitation x Dispersal) | 28 | 308.91 | 11.033 | 2.69 | **0.001** |
| Residual | 61 | 250.42 | 4.1053 |  |  |
